# Bioenergetics adaptations and redox homeostasis in pregnancy and related disorders

**DOI:** 10.1007/s11010-021-04215-0

**Published:** 2021-07-01

**Authors:** Lissette Sanchez-Aranguren, Sarah Nadeem

**Affiliations:** College of Health and Life Sciences, Aston Medical School, Aston University, Birmingham, UK

**Keywords:** Pregnancy, Mitochondria, Mitochondrial dysfunction, Mitochondrial-targeted drugs

## Abstract

Pregnancy is a challenging physiological process that involves maternal adaptations to the increasing energetics demands imposed by the growing conceptus. Failure to adapt to these requirements may result in serious health complications for the mother and the baby. The mitochondria are biosynthetic and energy-producing organelles supporting the augmented energetic demands of pregnancy. Evidence suggests that placental mitochondria display a dynamic phenotype through gestation. At early stages of pregnancy placental mitochondria are mainly responsible for the generation of metabolic intermediates and reactive oxygen species (ROS), while at later stages of gestation, the placental mitochondria exhibit high rates of oxygen consumption. This review describes the metabolic fingerprint of the placental mitochondria at different stages of pregnancy and summarises key signs of mitochondrial dysfunction in pathological pregnancy conditions, including preeclampsia, gestational diabetes and intrauterine growth restriction (IUGR). So far, the effects of placental-driven metabolic changes governing the metabolic adaptations occurring in different maternal tissues in both, healthy and pathological pregnancies, remain to be uncovered. Understanding the function and molecular aspects of the adaptations occurring in placental and maternal tissue’s mitochondria will unveil potential targets for further therapeutic exploration that could address pregnancy-related disorders. Targeting mitochondrial metabolism is an emerging approach for regulating mitochondrial bioenergetics. This review will also describe the potential therapeutic use of compounds with a recognised effect on mitochondria, for the management of preeclampsia.

## Introduction

Mitochondria are cellular organelles involved in the production of energy to support cell growth and proliferation. Recently, mitochondria are not only recognised as biosynthetic organelles but as important mediators in cell signalling pathways [1]. In the context of physiological processes such as pregnancy, the progress of placentation and foetal development require large amounts of energy and the mitochondria are key to sustain these increased metabolic demands. Besides, the maternal tissues are expected to adapt to these highly energetic events and to promote effective energy supply to the maternal-foetal interface.

Pregnancy encompasses physiological changes mainly driven by the placenta. However, the way the maternal tissues’ response to these demands is argued to be involved in the progression of a successful pregnancy. At early stages, usually during the first two trimesters, pregnancy allows the deposition of lipids in maternal tissues. This period is noticed as an “anabolic phase” [2] characterised by an increase in maternal fat storage [3, 4] and progressive decrease in fasting glucose levels while pregnancy advances linked to a 10% reduction in insulin sensitivity as compared with pregravid estimates [5]. Interestingly, although fasting glucose levels are reduced, hepatic glucose production (through gluconeogenesis and glycogenolysis) is increased, leading to an increase in fasting insulin. Consequently, the decrease in maternal hepatic insulin sensitivity results in enhanced hepatic glucose production [5]. Towards late gestation, the maternal metabolic status is characterised by a “catabolic phase” in where the peripheral insulin sensitivity is further reduced and peritoneal and subcutaneous fat storage is broken-down serving as a calorie source for mother and foetus [2, 5]. These events are so far well described and demonstrate the existence of active variations in the energetic requirements throughout pregnancy.

The maternal endothelium can respond to cellular signals from the mother and the foetus and these adaptations may involve a fine-tuned regulation of the by-products of metabolic pathways. Research suggests that both, placenta and maternal endothelium are highly energetic tissues using oxygen to produce energy through oxidative phosphorylation (OXPHOS) via mitochondria. This process also supports reactive oxygen species (ROS) formation that regulates intracellular signalling and tissue adaptations [6, 7]. ROS are increasingly recognised as signalling molecules regulating a myriad of physiological processes [7, 8]. Nevertheless, an imbalance in the cellular production and antioxidant defences [9], known as oxidative stress, triggers various cellular events that disturb signalling pathways leading to the onset of oxidative stress-related conditions [10].

So far, oxidative stress has been implicated as a mediator in the pathophysiology of a variety of pregnancy-related disorders, such as preeclampsia, intrauterine growth restriction (IUGR) and gestational diabetes [11–14]. Therefore, understanding the molecular mechanisms involved in the redox homeostasis during pregnancy is key to identify therapeutic targets that could potentially address these gestational disorders. This review aims to describe the role of mitochondria to sustain and to adapt to the high metabolic demands imposed by pregnancy, focusing on identifying the metabolic fingerprint of placental mitochondria at different stages of pregnancy and recognising the mitochondrial perturbations associated with pathogenic outcomes in maternal tissues. Emerging evidence suggests that targeting mitochondrial metabolism in pregnancy might be of therapeutic interest. Therefore, this review also summarises current mitochondrial-targeted drugs and their observed effect on the progression of gestation and outcomes associated with preeclampsia.

## Mitochondria: bioenergetics and signalling organelles

Mitochondria play a key role in the production of energy in eukaryotic cells. These double membrane-bound organelles support most of the cellular energetic demands by generating adenosine triphosphate (ATP) [15]. Also, mitochondria generate ROS, regulate cytosolic calcium levels and modulate apoptosis [16].

**Fig. 1:**
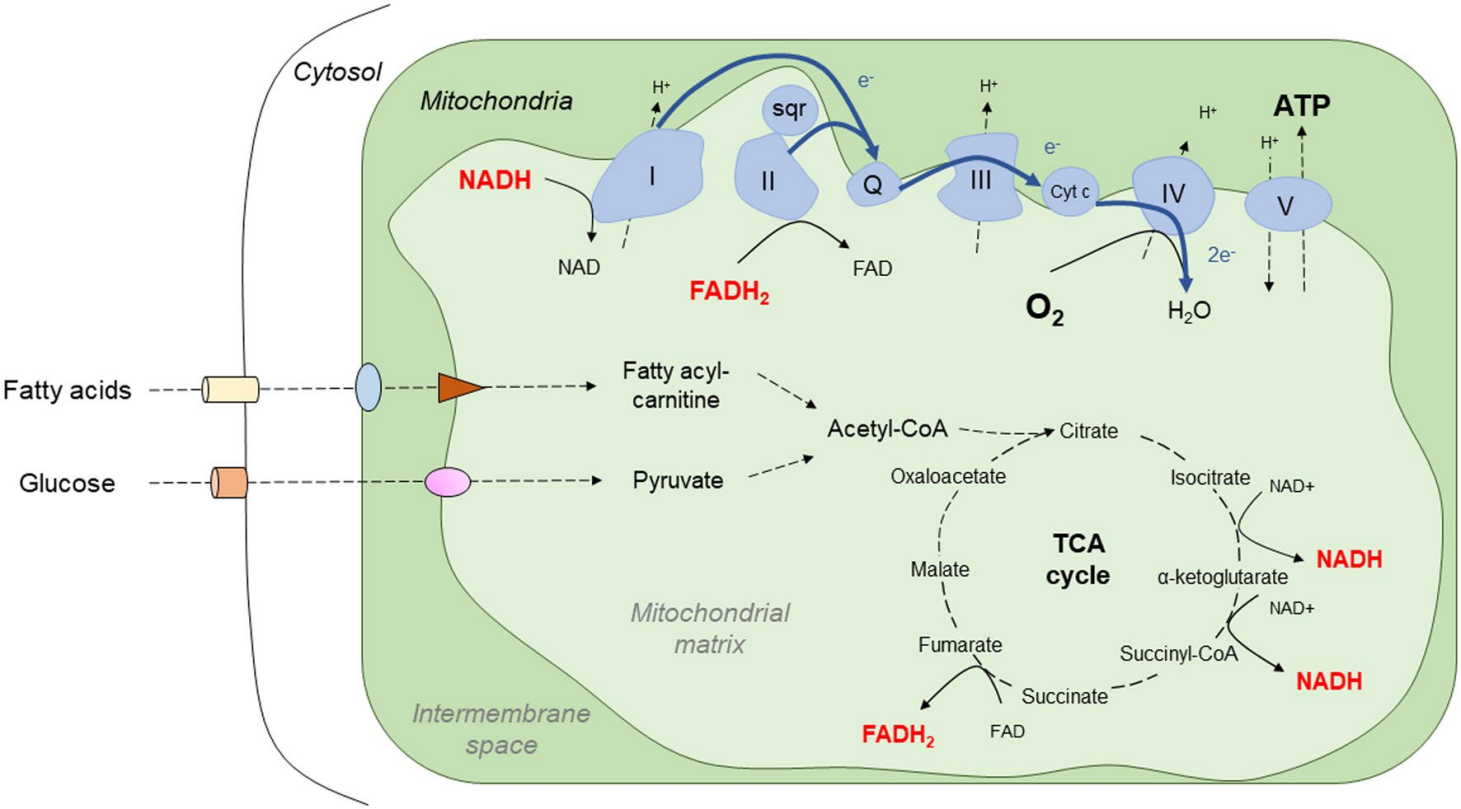
Schematic overview of mitochondrial bioenergetics of the electron transport chain and the tricarboxylic acid cycle. H^+^, hydrogen; sqr, sulfide quinone oxidoreductase; Q, coenzyme Q; Cyt c, cytochrome c; e^−^, electron

From the bioenergetics perspective, mitochondria are responsible for two major processes, the production of ATP and the generation of metabolic intermediates [17]. The mitochondrial production of ATP relies on the oxidation of metabolic substrates by the tricarboxylic acid cycle (TCA) and the electron transport chain (ETC) in the presence of oxygen. The TCA cycle generates metabolic intermediates and reducing equivalents that would feed the ETC and serve as building blocks for macromolecule biosynthesis [17]. Both, the TCA cycle and the ETC are tightly coordinated as the oxidation of reducing equivalents, NADH and FADH_2_, are required for the TCA [17, 18] (Fig. 1).

The mitochondrial oxidation of substrates requires oxygen as the last acceptor of electrons in the ETC [19]. Therefore, the mitochondria are one of the most important sites for ROS formation resulting from the reduction of molecular oxygen to produce superoxide. The production of mitochondrial reactive oxygen species (mtROS) is strongly regulated by antioxidant enzymes such as superoxide dismutase (SOD) that convert superoxide to hydrogen peroxide (H_2_O_2_). Several enzymes including peroxiredoxins, glutathione peroxidases and catalase remove H_2_O_2_ and hence, regulate intracellular ROS levels [7]. In biological systems, mtROS are known to play a crucial role in the adaptation to different stimuli including hypoxia, cytokine stimulation and calcium influx [7]. Although the redox biology of pregnancy and associated complications remain largely unexplored, evidence suggests that dysregulation of mtROS homeostasis causes mitochondrial dysfunction and oxidative stress and these events are associated with the onset of adverse gynaecological outcomes [20].

### The mitochondria during pre-implantation and early development

The mitochondria are the most abundant cytoplasmic organelles in oocytes. These organelles experience substantial changes during preimplantation development to provide for the energetic requirements of the embryo and participate in key signalling cellular pathways [21, 22]. The mature oocyte contains large amounts of mitochondria accounting for approximately 23% of its volume [23]. It is the mature oocyte that provides with the mitochondrial cargo for the embryo and although the spermatozoa do not provide with mitochondria, these organelles are important for sperm motility and male fertility [24]. After implantation, the blastocyst experience a significant metabolic shift with enhanced reliance on glycolysis for ATP production [25]. Still, it has been reported that OXPHOS contributes to the generation of ROS production during embryo development [26]. These observations indicate that mitochondria are crucial organelles of major importance for the production of mitochondrial-derived ROS participating in cell signalling events during embryogenesis.

### Placental mitochondria at early pregnancy

The development of the placenta initiates at embryo implantation, followed by the migration of trophoblast cells into the maternal decidua and the invasion and remodelling of maternal spiral arteries [27]. Several cell types compose the human placenta. In particular, the villous trophoblast cell linage, predominantly cytotrophoblasts (CT) and the syncytiotrophoblast (ST) are involved in key placental functions [28]. The invasion and remodelling of spiral arteries occur in an environment of low oxygen tension, yet, the recurrent invasion provides increased blood perfusion and oxygen to the placenta. This theory has been proven by polarographic electrode measurements in vivo, where it has been shown that the foetal-placental oxygen tension at 8–10 weeks of gestation is approximately 17.9 ± 6.9 mm Hg while at 12–13 weeks the oxygen tension increases to 60.7 ± 8.5 mm Hg [29]. These observations also suggest that variations in oxygen bioavailability are a normal feature of healthy pregnancies. Besides, it is inferred that the increased availability of oxygen may promote OXPHOS and the formation of physiological levels of ROS. Supporting this evidence, the role of hypoxia in the differentiation of CT has been well documented. Low oxygen (2% oxygen) promotes the differentiation of isolated first trimester CT into extravillous trophoblasts (EVT) and inhibits the differentiation into ST [30].

Variations in oxygen levels in the placenta disturb the function, dynamics and integrity of the placental mitochondria. At 9 weeks of gestation, exposure of ST to 21% oxygen significantly deteriorates the mitochondrial integrity resulting in swollen mitochondria displaying irregular shapes and degeneration of their cristae. Conversely, when maintained at low oxygen tension, the ST mitochondria preserve their regular shape and condensed state with clearly defined cristae [31]. Although ST contains abundant mitochondria [31], the expression of antioxidant enzymes copper- and zinc-containing superoxide dismutase (Cu/ZnSOD) [32] and catalase [33] are scarcely detected by immunohistochemistry. In contrast, CT show high expression of these antioxidant enzymes [32, 33]. These striking differences in oxygen sensitivity suggest that ST might be physiologically protected for low oxygen tensions in vivo. However, a sudden burst of oxygen bioavailability would lead to increased vulnerability to oxidative stress. From the bioenergetics perspective, an increased vulnerability to oxygen availability in an environment of low oxygen may diminish the reliance of ST on mitochondria to sustain metabolic processes driven by mitochondria.

**Fig. 2:**
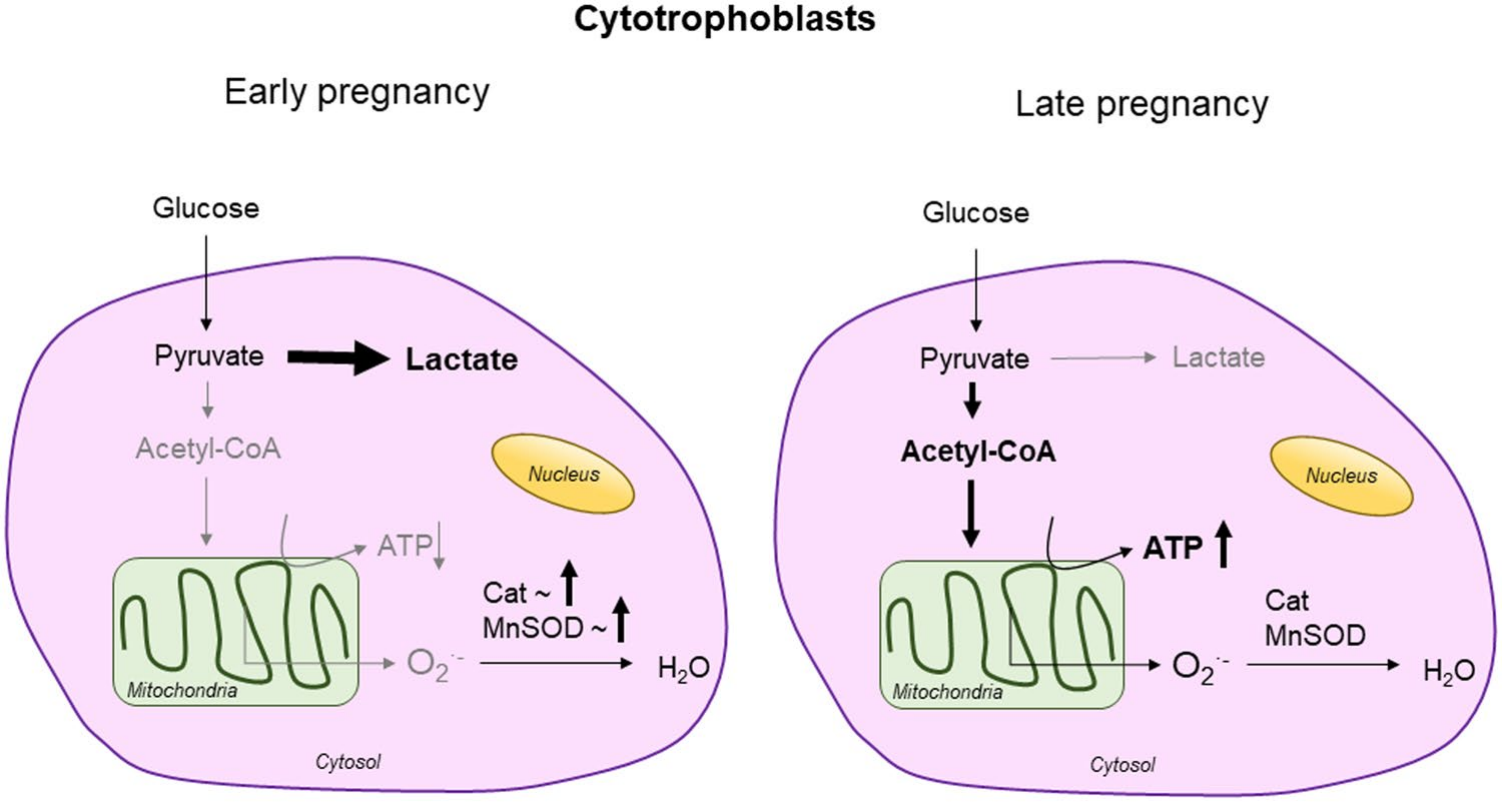
Proposed mechanisms of cellular bioenergetics in cytotrophoblasts at early and late stages of pregnancy. Cat, catalase; MnSOD, manganese superoxide dismutase

As pregnancy progresses, there is a continuous differentiation of CT to multinuclear ST which allows the formation of the outer layer of the placental villi. This process of differentiation relies on changes in mitochondrial energy production and signalling interactions [34, 35]. A significant shift in energy metabolism precedes trophoblast fusion in CT differentiation to ST, including increased lactate production with enhanced anaerobic pathways [36] and lower antioxidant capacity [37] (Fig. 2).

So far, several studies have put in evidence that CT and ST display strong differences in the structure and function of their mitochondria. However, our understanding of the role of mitochondria to sustain the energetic demands of EVT is limited. In the attempt to better understand the importance of mitochondria in EVT metabolism, a study has shown that the well-established immortalised EVT cell line (named HTR-8/SVneo) are resistant to apoptosis when exposed to low oxygen environment [38]. Interestingly, when cultured in normal oxygen tension, the EVT show an increased reliance on glycolytic pathways and suppressed mitochondrial reserve capacity. The EVT displays a highly glycolytic metabolism and therefore, are less sensitive to mitochondrial impairments associated with anti-angiogenic factor signalling [39].

Nevertheless, because of the variations in oxygen bioavailability, ROS are produced. However, these molecules have demonstrated to play important physiological signalling roles in early stages of gestation. For example, ROS triggers vascular endothelial growth factor signalling cascade and activates glucose transporters promoting the process of angiogenesis [40]. Although several studies suggest that ST and EVT are less reliant on mitochondrial function for energetic purposes, their mitochondrial machinery is fully capable of the generation of mtROS. Consequently, the role of mitochondria in the early stages of pregnancy might be mainly focused on mtROS production to promote cell signalling pathways and angiogenesis, allowing foetal growth and development.

These observations suggest that early in pregnancy, instead of exhibiting a crucial role in the generation of energy, mitochondria might be responsible for signalling interactions that could result in successful placental adaptations to stress (i.e. low oxygen, nutritional deprivation).

### Late pregnancy: role of placental mitochondria

Several studies have identified the response of term placental trophoblasts to physiological and pathological stimuli. Nevertheless, the morphological structure and functionality of isolated term trophoblasts do not resemble the nature of trophoblasts at the early stages of pregnancy. Currently, the vast majority of trophoblast physiology research, although elegantly executed, employ term trophoblasts [41–43]. Considering that the placenta is discarded after birth as waste, probably the easiest approach to study placental function is utilizing term placental cell isolation and ex vivo studies.

During the last trimester of gestation, the layer of CT progressively disappears [44], the population of EVT is partially replaced by fibrinoid structures and at term, only a thin layer of ST covers the chorionic villi [45, 46]. Several reports suggest that ST are mainly responsible for the metabolic activity of the maternal-foetal unit, as they are in intimate contact with the maternal blood. However, throughout gestation, CT are responsible for re-generating the layer of ST, which suggests that these trophoblasts might possess higher metabolic requirements. Likewise, CT highly expresses genes involved in the regulation of lipid uptake and metabolism [46] and displays higher glycolytic and mitochondrial activity in comparison to the differentiated term ST [41].

Studies evaluating the mitochondrial dynamics associated with the differentiation of term CT to ST have evidenced fragmentation of the mitochondrial network resulting in clear differences in the mitochondrial structure of term trophoblasts populations [41]. Term CT mitochondria are relatively larger with lamellar cristae whereas ST mitochondria are smaller, dense and with vesicular cristae [28, 47, 48]. This structural dissimilarity has been related to defective dimerization of mitochondrial complex V (ATP synthase) in ST [35]. Consistent with these structural differences, proteomic analysis in isolated term placental mitochondria demonstrated a differential regulation of 651 proteins with 29 of those being statistically different between ST and CT. Twenty-four of these proteins, including ATP synthase subunits α and β, superoxide dismutase and phosphoenolpyruvate carboxykinase were downregulated in ST whereas only five proteins were upregulated [49]. These variations imply that term trophoblast subpopulations; e.g. CT and ST may possess a distinguishable and unique metabolic phenotype. Supporting this assumption, studies using real-time bioenergetic assays have shown that CT display significantly higher levels of basal, ATP coupling and non-mitochondrial oxygen consumption rates when compared to ST, with no significant change in maximal respiration and reserve respiratory capacity [42]. Besides, it has been shown that the generation of ATP is increased in CT in comparison to subpopulations of ST [43]. Together, these observations suggest that term CT exhibit higher mitochondrial activity than ST. From the metabolic point of view, term CT displays different features than ST that may allow us to distinguish both trophoblasts subpopulations.

The role of placental mtROS in modulating cell signalling processes has been also addressed in trophoblast-like cells. Studies by Walker et al. using BeWo trophoblasts have identified that exposure to rotenone (mitochondrial complex I inhibitor) resulted in the generation of mtROS, reduced cellular fission and increased mitochondrial fragmentation, determined by decreased expression of mitochondrial fusion regulating proteins: mitofusin 2 (MFN2) and optic atrophy protein 1 (OPA1) and increased the expression of fission regulating protein dynamin-related protein 1 (DPR1) in differentiated and undifferentiated BeWo cells. Rescue experiments using pre-treatment with antioxidant N-acetyl cysteine showed normalisation of MFN2 and OPA1 and partial restoration of DPR1 mRNA expression. These observations highlight the importance of mitochondrially-derived ROS in sustaining the mitochondrial structure and function of placental cells [50].

### Apoptosis in pregnancy

Apoptotic cell death is a dynamic process by which dysfunctional cells are removed to maintain normal tissue function. It can be initiated through the mitochondria via the intrinsic pathway, in response to cellular stressors such as DNA damage [51]. The process is activated by the tumour suppressor protein, p53 which leads to transactivation of the proapoptotic Bcl-2 family [52]. Cytochrome c binds to apoptosis protease activation factor-1 resulting in oligomerisation and apoptosome formation [53]. The apoptosome facilitates caspase activation, specifically the recruitment of caspase 9 which activates caspase 3 and caspase 7, leading to immunosilent cleavage of unwanted cells.

Programmed cell death is important in normal pregnancy for processes including implantation and healthy development of the placenta. A study by Galan et al. showed that in the preattachment phase of implantation, the developing blastocyst averts apoptosis from human endometrial epithelial cells in order to adhere to the endometrium. This was indicated by a reduction in human endometrial epithelial cells apoptotic cells (35.2%) compared to 48.8% for human endometrial epithelial cells cultured without the blastocyst. However, this study showed a considerable increase in paracrine apoptosis after adhesion which suggested that the regulation of apoptosis is a key process at early stages of pregnancy [54]. The role of apoptosis in pregnancy has shown that the development of normal pregnancies is associated to an increased rate of maternal peripheral blood apoptosis [55]. However, in terms of pregnancy, it has been shown that pregnancy itself is linked to a reduced rate of neutrophil apoptosis, in comparison to non-pregnant women, suggesting an explanation for the neutrophilia evidenced in normal pregnancies [56]. In terms of placental apoptosis, studies show that apoptosis increases through gestation and significantly accelerates at term [57]. Interestingly, increased levels of placental apoptosis have been identified in several pregnancy pathologies [56], including preeclampsia [58, 59] and IUGR [60, 61]. However, the molecular mechanisms associating exaggerated apoptosis with pregnancy complication remains unclear.

## Mitochondrial adaptations through pregnancy

### Cardiovascular system

Mitochondria consist of about 30% of the volume of cardiomyocytes and accounts for the bioenergetic homeostasis of the heart [62]. Some of the most important pregnancy-related maternal adaptations occur in the cardiovascular system. Those maternal hemodynamic changes of pregnancy allow increased blood flow to various organs to meet energetics demands [63, 64], leading to vasodilation of the systemic vasculature, increased cardiac mass (∼ 50%), increased cardiac output (∼ 20–50%) resulting in increased energetic demands [65] and by the end of the first trimester of pregnancy a 50% increase of the glomerular filtration rates in kidneys [66, 67].

Although pregnancy requires a remarkable adaptation of the cardiovascular system to provide blood and metabolites to maternal organs and the foetal-placental unit, only a few studies in humans describe the metabolic adaptations of the heart during a normal pregnancy. Interestingly, during pregnancy, cardiac mitochondria can adapt to energetic challenges by increasing the rates of mitochondrial substrates utilisation. In this regard, studies in rats have shown that the rates of glucose utilisation decrease 7-fold as the pregnancy progresses while rates of fatty acid and ketone bodies utilisation increase by approximately 2.5-fold and 6-fold, respectively [68]. These differences in substrate preference may imply that cardiac mitochondrial metabolic adaptations are an important hallmark in the progression of normal pregnancies. This evidence suggests that early pregnancy-cardiac metabolism is mainly glucose-driven while towards later stages of gestation, mitochondria appear to play a key role in supporting the bioenergetic homeostasis in the heart.

Maternal metabolic impairments in gestational diabetes and obesity, increase the risk of the offspring to develop heart disease later in life [69]. Studies in neonatal rat cardiomyocytes showed that offspring from normal diet-exposed dams have highly dynamic mitochondria while diabetes or high-fat diet-exposed rats resulted in offspring’s neonatal cardiomyocytes with shorter and wider structures along with defective gene expression of mitochondrial fusion regulating proteins: mitofusin 1 (MFN1), MFN2, OPA1 and pro-fission DRP1, mitochondrial fission regulating proteins: mitochondrial fission factor (MFF) and mitochondrial fission process 1 (MTFP1). Interestingly, these differences are influenced by foetal sex [70]. These observations infer that those cardiac mitochondrial perturbations arising in metabolically impaired pregnancies, may be involved in the onset of cardiovascular disease in the offspring at later stages of their life.

### Renal system

Several pregnancy pathological complications and co-morbidities are associated with kidney-related impairments [71]. Studies by Popkov et al. showed that pregnancy enhances the mitochondrial membrane potential in renal isolated mitochondria in a model of renal ischaemia/reperfusion injury in rats [72]. In vivo models of preeclampsia have shown an association with excessive mtROS production and pathological outcomes. The reduced uteroplacental perfusion (RUPP) model established in rats showed that ischaemia insults impair the renal mitochondrial function by reducing the expression of mitochondrial complexes I and II, suppression of mitochondrial respiratory parameters [73] exacerbated production of mtROS [74]. Also, a model of hypertension in pregnancy in rats exposed to autoantibodies to the angiotensin II type 1 receptor, evidenced increased production of H_2_O_2_ as an indicator of oxidative stress [75]. In general, these studies are supportive of the existence of oxidative stress in renal tissue from complicated pregnancies and suggest that modulation of the oxidative damage in the kidney may be of therapeutic interest when managing these disorders.

### Skeletal muscle

The skeletal muscle accounts for approximately 50% of the body mass and its energetic requirements rely on glucose and fatty acid utilisation [76]. Although there are numerous studies addressing the role of mitochondria in human skeletal muscle during excersice, our understanding of the role of mitochondria to sustain skeletal muscle function in phases of human reproduction is not yet clear. The effects of low maternal energy diets have been studied in pigs, showing that nutritional deprivation reduces the mitochondrial DNA (mtDNA) copy number, citrate synthase and NAD^+^-to-NADH ratio in their offspring’s skeletal muscle compared to standard energy diet offspring. This restrictive energetic diet caused a reduction in the transcription of mitochondrial biogenesis genes, PPARG Coactivator 1 Alpha (PPARGC1-α) and Sirtuin 1 and impaired the antioxidant defences expressed by reduced SOD and catalase mRNA and protein expression in foetal skeletal tissue [77]. The effects of maternal nutritional restriction have been associated with the onset of several pregnancy-related disorders [78] and the programming of foetal developmental alterations [79]. Therefore, restrained maternal nutrition might seriously alter the skeletal muscle homeostasis in the offspring and these events are likely to be influenced by defective mitochondrial biogenesis signals reprogrammed in the offspring. Nevertheless, most studies attempting to reveal the role of mitochondrial dysfunction in skeletal muscle during pregnancy, have been performed using animal models, requiring a more attentive interpretation of those implications for human pregnancy.

## Mitochondrial dysfunction in pregnancy-related disorders

The study of the dysregulation in mtROS production in pregnancy-associated complications such as; preeclampsia, IUGR, gestational diabetes and pre-term birth is a hot topic of research in the field of reproductive biology. There is accumulative evidence demonstrating the effectiveness of mitochondrially targeted therapeutics to modulate the redox balance in a variety of these disorders. Hence, this section summarises key mitochondrial disturbances observed in pathological pregnancy outcomes including studies in placental, vascular and cardiovascular tissues and highlights the potential benefit of drugs with recognised effects on mitochondria to potentially manage preeclampsia.

### Preeclampsia

Preeclampsia is a disorder of pregnancy arising from the 20^th^ week of gestation, clinically characterised by newly-onset hypertension and proteinuria [80]. From the molecular point of view, preeclampsia is associated with the upregulation of circulating levels of anti-angiogenic factors soluble Flt-1 (sFlt-1) and soluble endoglin [81] that might account for maternal vascular dysfunction and oxidative stress described in preeclampsia [78].

The role of the placental mitochondria in preeclampsia has been well studied. However, there are inconsistencies in results showing dramatic variations in terms of mitochondrial structure, content and function. Human studies have shown that gene expression of mitochondrial dynamics proteins, OPA1, fission 1 (FIS1), MFN1 and MFN2 are impaired in preeclamptic term placentas [82, 83]. Conversely, others have shown elevated expression in markers of mitochondrial fission; MFN1 and MFN2 in preeclampsia [84]. The placental mitochondrial content and mitochondrial biogenesis signals, important for the preservation of tissue function and metabolic activity, have also shown some inconsistencies [85]. In this regard, while some have reported decreased OXPHOS activity [86] and reduced expression of mitochondrial ETC complexes in preeclampsia [87–89], a recent report demonstrate otherwise [82]. One potential explanation for these diaparities can be attributed to the gestational age of preeclampsia onset. For example, studies by Holland et al. showed that pre-term preeclamptic placental display a reduction in mitochodnrial antioxidant activity (SOD activity in mitochondrial content) when compared to normotensive pre-term, term normotensive and term preeclamptic counterparts [82]. Another potential explanation can be attributed to the severity of preeclampsia included in those studies. In this regard, studies by Zhou et al. focused on term preeclamptic placentas, including severe preeclapmsia patients [84]. As these variables on preeclampsia development (gestational age and severity) can be crucial for diagnose and management [90, 91], a vigilous interpretation of results is suggested in order to better understand the role of mitochondria in the onset of this complication.

Others have reported evidence of dysregulation in the mitochondrial integrity and function in models displaying preeclampsia-like signs. Several mice models mimicking the preeclampsia condition induced by treatment with Nw-nitro-L-arginine-methyl ester (L-NAME, nitric oxide synthase inhibitor), lipopolysaccharide, β2 glycoprotein I [92], sFlt-1-injected mice [93] and STOX1 transgenic mouse [94], have shown similar impairments in the structure of trophoblast mitochondria exhibiting increased swelling and cristae disappearance. Similarly, the expression of mtROS detoxifying enzymes, including the mitochondrial uncoupling protein 1 (UCP-1) and superoxide dismutase 2 (SOD2) are reduced in models of preeclampsia [74, 94].

As preeclampsia originates from the second trimester of pregnancy, studying term placental tissue might not be a reliable approach to either study molecular mechanisms of the disorder or to evaluate effective therapeutics to prevent it. Given that preeclampsia is a maternal complication, mostly affecting the vasculature of the mothers, i*n vitro* studies using endothelial cells have provided new insights into the contribution of dysfunctional mitochondria in vascular dysfunction. Exposure of endothelial cells to plasma from preeclamptic women have shown to significantly increase the production of mtROS [39, 73, 95] and to suppress the basal and maximal respiratory capacity [39, 95]. The expression of mitochondrial respiratory complexes, citrate synthase and fatty acid oxidation is reduced in human umbilical vein endothelial cells (HUVEC) isolated from preeclamptic pregnancies [96]. Studies in rats emulating the preeclampsia-like disorder have also supported the role of mitochondria in the production of mtROS and suppression of OXPHOS in renal tissue [73, 74, 97]. Likewise, analysis of blood samples from preeclamptic and uncomplicated pregnancies showed increased superoxide generation that negatively correlates with microvascular endothelial function in preeclampsia [12].

### Intrauterine growth restriction (IUGR)

IUGR is a pregnancy-specific disorder characterised by a reduced foetal growth rate in comparison to the expected growth at a specific stage of gestational development [98]. The molecular features of IUGR share a common placental phenotype with other hypertensive disorders of pregnancy, characterised by “placental insufficiency” at the early stages of pregnancy [99]. The role of mitochondrial dysfunction in IUGR pathogenesis is not yet clear. However, there is evidence suggesting that mitochondrial impairments are associated with this complication [100].

Studies in placental tissue from IUGR patients showed an increased mitochondrial content (measured as mtDNA) in this group in comparison to normal pregnancies [99, 101]. Interestingly, the content of mitochondria in isolated placental CT is lower in the IUGR group [99]. Similarly, mRNA levels of mitochondrial biogenesis regulator, nuclear respiratory factor 1 (NRF1) is increased in placentas from IUGR but reduced in isolated CT. The expression and activity of mitochondrial complexes subunits are also reduced in IUGR [99, 102]. Nevertheless, controversial results have been reported showing increased OXPHOS efficiency in comparison to control [99] while others have described the mitochondrial activity to be reduced [102]. A potential explanation for these contrasting results may be associated to the complexity of the placental structure and multiple cell types composing the placenta. For example, cells composing the maternal section include trophoblasts, stromal and fibroblast-like cells and macrophages. In contrast, the foetal section is composed by a layer of ephitelial cells resting over a layer of connective tissue associated with foetal blood vessels [103]. Therefore, results found in whole placental tissue could be related to cell types other than CT.

To explain these variations in mitochondrial content and activity, Jones et al. suggested that these might result as a metabolic compensatory mechanism. In this regard, placentas from IUGR pregnancies have shown to express increased mRNA levels of glycolysis-regulatory gene PDK1 (Pyruvate Dehydrogenase Kinase 1) [104]. These exciting results suggest that other cell types, including ST, mesenchymal cells and fibroblasts might contain a significant number of mitochondria and may account for the increased content observed in whole placental tissue from IUGR. Knowing that in normal pregnancies the mitochondrial content and structure of ST tend to reduce while pregnancy progresses, these observations suggest that IUGR may accompany a dysregulation in placental mitochondrial adaptations along with modulation of less efficient pathways such as glycolysis, possibly to sustain the enhanced energetic requirements exert by the foetus.

Plasma mediators in IUGR pregnancies have been shown to reduce endothelial cell viability and to promote intracellular ROS production in vitro. Electron microscopy studies have revealed that HUVEC isolated from IUGR pregnancies evidence cellular abnormalities including mitochondrial swelling, enlarged intermembrane spaces and disrupted cristae [105]. These observations reveal a similar pattern with preeclampsia-induced mitochondrial dysfunction in endothelial cells, suggesting a common pathway in the pathophysiology of these disorders. In a rat model of IUGR, it was evidenced a reduction in pyruvate, succinate and α-ketoglutarate oxidation rates along with increased manganese superoxide dismutase (MnSOD) protein levels [106]. IUGR is also associated with skeletal muscle perturbations linked to defective glucose homeostasis and reduced muscle respiration [100].

Other evidence suggests that mitochondrial dysfunction is a mechanism of foetal metabolic programming in offspring from IUGR. In a model of IUGR established in pigs, it was shown that IUGR offspring fed with a high-fat diet exhibit reduced activity of lactate dehydrogenase (LDH) and glucose-6-phosphate dehydrogenase (G6PD) accompanied by suppressed succinate and glutamate-induced OXPHOS activity, reduced mitochondrial contents and downregulation of mRNA expression of genes involved in mitochondrial biogenesis in skeletal muscle [107]. Similarly, a model of IUGR in mice established by maternal undernutrition, affected the cardiac bioenergetics by suppressing fatty acid oxidation [108], suggesting a greater susceptibility of IUGR offspring to dysregulation in cardiac energetic balance.

### Gestational diabetes (GDM)

The first detection of hyperglycaemia during pregnancy is classified as “diabetes mellitus in pregnancy” or “gestational diabetes (GDM) mellitus” by the World Health Organization (WHO) [109]. In some circumstances, maternal tissues fail to sustain the metabolic adaptations required for pregnancy resulting in complications such as GDM [110, 111]. Although the molecular events leading to GDM are still not well understood, the dysregulation of mitochondrial substrate oxidation has been proposed to play a role in GDM.

Studies in term trophoblasts from women with GDM showed downregulation of mitochondrial biogenesis modulator, PGC1-α (peroxisome proliferator-activated receptor gamma coactivator 1-alpha) and suppressed parameters of mitochondrial function along with a two-fold increase in glucose transporter GLUT-1 expression [112]. Mitochondrial dynamics are also impaired in GDM showing pro-fusion features with elevated OPA1 and DRP1 expression [113]. GDM is also linked to increased placental lipid peroxidation and oxidised proteins (carbonyls) [114]. In the maternal skeletal muscle, GDM has also shown to impair the expression of mitochondrial complex I [115]. These observations propose that the metabolic perturbations exerted by GDM in the placental tissue may result in the inability of maternal tissues to adapt to the metabolic demands.

When a healthy diet along with exercise is not enough to regulate maternal glucose levels, drugs such as insulin and metformin are indicated to manage GDM [116]. However, metformin is known to suppress the activity of mitochondrial complex I and to prevent complex I-induced mtROS generation [117]. As the American College of Obstetricians and Gynaecologists (ACOG) and the National Institute for Health and Care Excellence (NICE) guidelines recommend, metformin represents the first line of choice for the management of GDM [118, 119]. Therefore, our interpretation of studies evaluating the function of mitochondria using tissues or cells derived from GDM patients treated with metformin should be cautious.

## Targeting mitochondrial metabolism in the management of preeclampsia

Antioxidant therapies have largely demonstrated to fail in the management of preeclampsia. One possible explication relies on the potential inability of these drugs to reach and accumulate in relevant cellular compartments, such as the mitochondria. In the past decades, compounds targeting the mitochondria have gained great interest in their abilities to moderate mtROS generation and cellular bioenergetics in a myriad of pathological disorders [120].

Some unique features on the mitochondrial inner membrane composition and function, such as its high transmembrane potential and particular phospholipid composition allowed the targeting of mitochondria [121]. Approaches for directing drugs for delivery and accumulation within the mitochondria include links to a lipophilic cation moieties such as triphenylphosphonium (TPP^+^), cardiolipin and heterocyclic aromatic cations [120].

Emerging research describes mitochondrial dysfunction and oxidative stress as molecular hallmarks of preeclampsia. Therefore, recent efforts to elucidate effective therapies to manage this disorder have focused on the mitochondria. Different drugs with effects on mitochondria (either specifically targeted or not) have been evaluated in models of preeclampsia. A list of compounds with recognised effects on mitochondrial function targeting the restoration of mitochondrial function in vitro, in vivo and clinical trials for the management of preeclampsia are described in Table 1.

**Table 1:** Drugs with recognised effect on mitochondrial function used in models of preeclampsia established in vitro, in vivo and clinical trials

	Study approach	Drug	Treatment and dose	Outcomes	Reference
Assessment					
HUVEC	Culture with (a) 3% plasma (b) 200 µM hydrogen peroxide	Mito-Tempo	Pre-treatment (2 h) with 5 µM	-Reduced mtROS generation -Reduced hydrogen peroxide induced-cell death	[95]
HUVEC	Culture with 10% serum from RUPP rats treated with mito-Tempo and mitoQ	Mito-Tempo MitoQ	Mito-Tempo: 1 mg/kg per day via osmotic pumps MitoQ: 500 µM via oral gavage	-Reduced cellular mtROS production	[73]
Human primary cytotrophoblasts	Culture in low oxygen conditions (5% O_2_)	AP39	Pre-treatment (30 min) with 10, 25 and 50 µM	-Reduced mtROS generation -Abrogation of sFlt-1 production	[129]
HUVEC, primary villous CT and placental villous explants		Metformin	1, 2 and 5 mM	-Reduction of sFlt-1 and soluble endoglin mRNA levels and protein expression	[123]
HUVEC, primary villous CT and placental villous explants		Metformin + sulfasalazine	200 μM metformin or 200 μM sulfasalazine alone or in combination	-Reduction in the secretion of sFlt-1 and soluble endoglin	[122]
HUVEC, primary villous CT and placental villous explants		Metformin + esomeprazole	1000 μM metformin or 25 μM esomeprazole alone or in combination	-Reduction in the secretion of sFlt-1 and soluble endoglin	[124]
First trimester and term placenta	Culture with monoclonal antiphospholipid antibodies	MitoQ	0.1, 1 and 10 μM	-Reduction of ROS production of explants	[134]
Sprague Dawley rats	RUPP	Mito-Tempo MitoQ	MitoTempo: 1 mg/kg per day via osmotic pumps MitoQ: 500 µM via oral gavage	-Reduced maternal blood pressure -Improved pup and placental weight	[73]
Pregnant Institute of Cancer (ICR) Research mice	RUPP	Mito Q	100 μM/Kg/day via oral gavage	-Administration at late stage of pregnancy (E13.5–17.5) resulted in alleviated preeclampsia-like signs and abrogation of oxidative stress in placenta -Administration in early stages (E7.5–11.5) elevated blood pressure, foetal growth restriction and proteinuria	[133]
Wistar rats	200 mg/kg/day L-NAME subcutaneous injection (5 days)	Coenzyme Q10	60 mg/kg/day via gavage	-Reduction of Systemic blood pressure and proteinuria on day 21 of gestation -Increased pup size and weight	[137]
Clinical trials					
NCT00300937	Participants: 235 women 118 women: Received coenzyme Q10 117 women: Received placebo	Coenzyme Q10	200 mg daily from 20th weeks of gestation until delivery	-Rate of preeclampsia in the coenzyme Q10 group (14.4%) vs placebo group (25.6%) *p* = 0.035	[136]
PACTR201608001752102	Recruiting	Metformin			[126]
NCT03717701	Recruiting	Metformin + esomeprazole			

*HUVEC* human umbilical vein endothelial cells; *CT* cytotrophoblasts; *mtROS* mitochondrial reactive oxygen species; *sFlt-1* soluble Flt-1; *O*_*2*_ oxygen; *ROS* reactive oxygen species, *L-NAME* Nw-nitro-L-arginine-methyl ester; *E* gestation from embryonic day

### Metformin

Metformin, although not a mitochondrial-targeted drug, has proven abilities to cause unspecific inhibition of the mitochondrial complex I [117]. For that reason, the use of metformin for treating and or preventing preeclampsia has been included in this section.

Studies using villous CT, preterm preeclamptic villous explants and primary endothelial cells, have shown that metformin alone or in combination with other drugs, significantly abrogates sFlt-1 and soluble endoglin production [122–124]. As previously described in section “preeclampsia”, sFlt-1 and soluble endoglin are anti-angiogenic factors implicated in the pathogenesis of preeclampsia. In a model of preeclampsia in mice fed with a high-fat diet, the use of metformin has also shown to improve maternal blood pressure and foetal outcomes [125].

A phase II trial study (currently undergoing) protocol was recently published. This study aims to evaluate the efficacy of metformin to treat preterm preeclampsia (trial number PACTR201608001752102) [126]. Nonetheless, previous studies, although not focused on a cohort of preeclampsia patients, have provided insights into the potential effects of metformin to prevent this disorder. Nevertheless, the approach of metformin for managing preeclampsia should be cautions as two clinical trials performed in the UK focused on obese and diabetic cohorts showed contradictive results regarding the effectiveness of metformin on the risk of preeclampsia [127].

### AP39

**Fig. 3:**
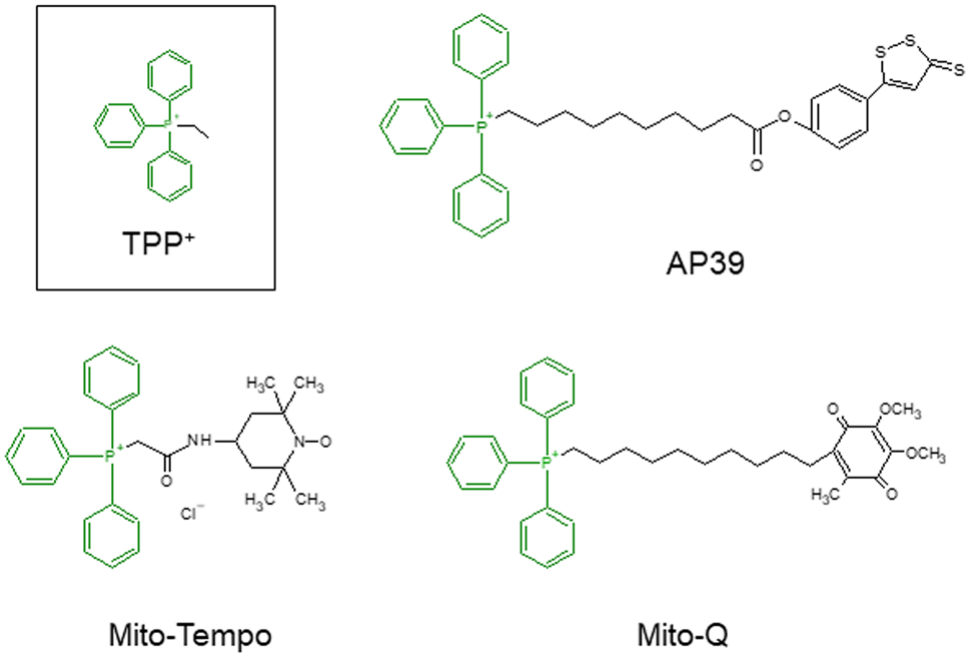
Structure of mitochondrial-targeted AP39, Mito-Tempo and Mito-Q. TPP^+^, triphenylphosphonium

The novel hydrogen sulfide (H_2_S) donor, AP39 consists of an H_2_S-donating moiety (dithiolethione) coupled to a TPP^+^ motif by an aliphatic linker targeting the release of H_2_S to the mitochondria (Fig. 3). The effectiveness of AP39 to promote the mitochondrial function has been tested in endothelial cells. This study showed that AP39 exerts cytoprotective effects and maintains the mtDNA integrity while improving the mitochondrial bioenergetics in endothelial cells exposed to high glucose levels [128]. Regarding the potential protective effect of AP39 on the placenta, Covarrubias et al. reported that AP39 significantly prevents sFlt-1 release, abrogates the generation of mtROS and enhances cytochrome c activity in isolated human primary trophoblasts exposed to hypoxia [129].

### Mito-Tempo and MitoQ

Mito-Tempo is a mitochondrial-targeted antioxidant compound consisting of the antioxidant piperidine nitroxide, superoxide dismutase mimetic (Tempo), linked to TPP^+^ [130]. MitoQ consists of an antioxidant compound (quinone) linked to TPP^+^ [131, 132] (Fig. 3). In a study in vitro, McCarthy et al. assessed the ability of mito-Tempo to protect against preeclampsia plasma mediators-induced mtROS generation in HUVEC. In this study, mito-Tempo also showed protective effects against H_2_O_2_-induced cell death [95].

In another study, both mitochondrial-targeted antioxidants suppressed the production of mtROS in HUVEC exposed to serum collected from RUPP animals. In this same report, the authors showed in an in vivo RUPP model that treatment with mito-Tempo and mitoQ reduce the mean blood pressure and improve pup and placental weight. However, mito-Tempo but not mitoQ improved litter size [73]. Similar reports have been provided by Yang et al. showing the effectiveness of mitoQ in alleviating preeclampsia-like signs in a RUPP model established in mice [133]. Studies in vitro, have shown that mitoQ partially prevents the production of ROS from placental explants exposed to monoclonal antiphospholipid antibodies [134].

### Coenzyme Q10

Coenzyme Q10 or ubiquinone is a lipid-soluble antioxidant that participates in the electron transfer from complexes I and II to complex III in the mitochondria [135]. The effectiveness of coenzyme Q10 to prevent the risk of preeclampsia was investigated in a double-blind randomised study. The study showed a significant reduction (*p* = 0.035) in the rate of preeclampsia in the coenzyme Q10 group in comparison to the placebo group [136]. Alongside, recent reports by Xu et al. have shown that coenzyme Q10 prevents preeclampsia-like signs in L-NAME treated rats [137].

## Conclusion

The role of mitochondria in pregnancy and its related complications have mainly focused on understanding the structural adaptations occurring in the mitochondria of subpopulations of placental trophoblasts at term. However, little is known about the behaviour of these organelles at the early stages of gestation. Through pregnancy, trophoblasts display structural and functional adaptations in their mitochondria, resulting in distinguished differences between subpopulations of trophoblast at term. These metabolic fingerprints might be accountable for the distinct function of trophoblasts to support and sustain the pregnancy. As subpopulations of term trophoblasts’ mitochondria behave differently, appropriate methods to study and compare the function of trophoblasts are required.

The onset of a myriad of pathological pregnancy outcomes is associated with oxidative stress arising at the early stages of pregnancy. Still, functional studies are mainly performed in isolated term trophoblasts and this approach is not representative of the developmental structures and characteristics of early trophoblasts. These limitations have hindered our understanding of the bioenergetics adaptations and redox systems at early stages and therefore have delayed the exploration of appropriate targets for effective treatments.

Many pregnancy disorders have been linked to placental insufficiency. However, it is equally important to explore the responses of the maternal endothelium and to recognise the capabilities of maternal tissues to sustain energetic requirements during pregnancy. In other scenarios, early metabolic perturbations are implicated in the onset of disease. In pregnancy, failure to adapt to increased energetics demands might lead to dysfunction in key maternal tissues, leading to adverse outcomes. Mitochondria are crucial organelles supporting energy production not only in the placenta but in the endothelium. Therefore, targeting mitochondria is an attractive approach to tackle a variety of oxidative stress-related disorders. Although its exploration is still novel, mitochondrial-targeted antioxidants have provided new insights for the effective management of preeclampsia in proof of concept studies. Recently, drugs such as metformin and coenzyme Q10 that although are not targeted to the mitochondria, are capable to exert mitochondrial effects and have been proposed to treat preeclampsia in clinical trials. It is still not clear whether mitochondrial-targeted drugs can prevent preeclampsia and more results are needed to clarify if the selective delivery of antioxidants and metabolic modulators to the mitochondria, are effective to prevent preeclampsia-like symptoms.
